# Transcriptional response in normal mouse tissues after i.v. ^211^At administration - response related to absorbed dose, dose rate, and time

**DOI:** 10.1186/s13550-014-0078-7

**Published:** 2015-01-28

**Authors:** Britta Langen, Nils Rudqvist, Toshima Z Parris, Emil Schüler, Johan Spetz, Khalil Helou, Eva Forssell-Aronsson

**Affiliations:** Department of Radiation Physics, Institute of Clinical Sciences, Sahlgrenska Cancer Center, Sahlgrenska Academy, University of Gothenburg, 413 45 Gothenburg, Sweden; Department of Applied Physics, Chalmers University of Technology, 412 96 Gothenburg, Sweden; Department of Oncology, Institute of Clinical Sciences, Sahlgrenska Cancer Center, Sahlgrenska Academy, University of Gothenburg, 413 45 Gothenburg, Sweden

**Keywords:** Astatine-211, Ionizing radiation, Normal tissue response, Radionuclide therapy, Biomarker

## Abstract

**Background:**

In cancer radiotherapy, knowledge of normal tissue responses and toxicity risks is essential in order to deliver the highest possible absorbed dose to the tumor while maintaining normal tissue exposure at non-critical levels. However, few studies have investigated normal tissue responses *in vivo* after ^211^At administration. In order to identify molecular biomarkers of ionizing radiation exposure, we investigated genome-wide transcriptional responses to (very) low mean absorbed doses from ^211^At in normal mouse tissues.

**Methods:**

Female BALB/c nude mice were intravenously injected with 1.7 kBq ^211^At and killed after 1 h, 6 h, or 7 days or injected with 105 or 7.5 kBq and killed after 1 and 6 h, respectively. Controls were mock-treated. Total RNA was extracted from tissue samples of kidney cortex and medulla, liver, lungs, and spleen and subjected to microarray analysis. Enriched biological processes were categorized after cellular function based on Gene Ontology terms.

**Results:**

Responses were tissue-specific with regard to the number of significantly regulated transcripts and associated cellular function. Dose rate effects on transcript regulation were observed with both direct and inverse trends. In several tissues, *Angptl4*, *Per1* and *Per2*, and *Tsc22d3* showed consistent transcript regulation at all exposure conditions.

**Conclusions:**

This study demonstrated tissue-specific transcriptional responses and distinct dose rate effects after ^211^At administration. Transcript regulation of individual genes, as well as cellular responses inferred from enriched transcript data, may serve as biomarkers *in vivo*. These findings expand the knowledge base on normal tissue responses and may help to evaluate and limit side effects of radionuclide therapy.

**Electronic supplementary material:**

The online version of this article (doi:10.1186/s13550-014-0078-7) contains supplementary material, which is available to authorized users.

## Background

In nuclear medicine, treatment using radiolabeled tumor-seeking agents is a vital option for patients with, e.g., metastases or inoperable tumors [1]. ^211^At is a synthetic α-emitter with metalloid and halogen characteristics and a mean linear energy transfer (LET) value that is nearly optimal for inducing double-strand breaks (DSB) in DNA [2,3]. As such, ^211^At has a high biological effectiveness for cell killing which renders it a promising candidate for radiolabeling in radionuclide therapy [3-7]. However, ^211^At can be liberated from a carrier molecule *in vivo* during degradation or metabolism and be retained in normal tissues [8-10]. Unbound ^211^At is accumulated in the thyroid gland through a mechanism similar to iodide resulting in high uptake and absorbed dose [9-15]. In other tissues, ^211^At uptake occurs at lower levels, although generally higher than for iodide, with tissue-specific differences leading to differential exposure to ionizing radiation throughout the body [8-10]. The increased uptake of ^211^At in non-thyroid tissues compared to, e.g., iodide increases the radiation risk and side effects in healthy tissues after treatment with ^211^At-labeled tumor-targeting agents. A detailed understanding of normal tissue responses is needed to establish accurate tolerance doses and thus to optimize treatment effectiveness. Basic research in nuclear medicine routinely uses nude mice to study tumor xenografts and, for instance, anti-tumor effects of targeted therapy. However, little is known about the quality and quantity of normal tissue effects *in vivo*, which is an important aspect for robust analysis and establishment of both effective and safe treatment parameters.

Many studies for biomarker discovery of ionizing radiation effects have been performed with *in vitro* model systems and external irradiation, predominantly γ-rays and X-rays [16,17]. In contrast, knowledge on basic tissue responses *in vivo* to internal radionuclide exposure is still scarce, specifically with regard to α-emitters and low absorbed dose. Within the framework of the European project Low Dose Research towards Multidisciplinary Integration (DoReMi), Pernot and colleagues summarized that ‘changes in RNA levels identified by transcriptomics’ had ‘unknown sensitivity’ and that ‘specificity to ionizing radiation and confounders (was) unknown at present time’ [18]. In previous studies on mice, we used intravenously administered ^211^At in the 0.064- to 42-kBq range and characterized genome-wide transcriptional responses after 24 h in various normal tissues: in thyroid [19] and in kidney cortex and medulla, liver, lungs, and spleen [20]. Radiation-induced cellular responses were demonstrated as complex and tissue-specific and to vary with absorbed dose level in a non-linear manner, which advises caution for extrapolation or interpolation of responses between absorbed dose levels [19,20]. Furthermore, comparatively few previously proposed ionizing radiation-associated genes from *in vitro* studies were differentially regulated, which demonstrated the need to identify biomarker genes in an *in vivo* setting. Despite pronounced differences in absorbed dose levels between thyroid and non-thyroid tissues, the total significant transcript regulation showed similar characteristics between all tissues, i.e., a distinct shift in regulation intensity between 0.64 and 1.8 kBq ^211^At. These findings suggested that responses in non-thyroid tissues were not only due to low-dose effects from ^211^At but also subject to systemic effects from, e.g., the ^211^At-accumulating thyroid gland [20]. These studies highlight the necessity to further expand the knowledge base on low-dose responses to ionizing radiation *in vivo* while regarding induced effects in a systemic and physiological context.

In this exploratory study, transcriptional gene expression responses were analyzed on a genome-wide scale using RNA microarray technology. Microarrays are a sophisticated method for both hypothesis building and hypothesis testing within the same experimental setup due to the vast amount and large scale of obtained data without limitation to a set of presupposed genes or regulatory pathways. However, to the best of our knowledge, only a few studies have been performed investigating basic transcriptional responses to internal radionuclide exposure to, e.g., ^131^I [21,22] or specifically ^211^At [19,20]. Furthermore, few robust molecular biomarkers for ionizing radiation exposure *in vivo* have been identified, since most studies have been performed *in vitro* [16,17].

The purpose of this study was to investigate genome-wide transcriptional responses over time in normal non-thyroid tissues following intravenous ^211^At administration in mice. The aim was to characterize tissue-specific transcriptome responses and to identify potential *in vivo* biomarkers for (very) low mean absorbed doses with sensitivity to dose rate.

## Methods

### Radionuclide production and radioactivity measurements

^211^At was produced via the ^209^Bi(α,2n)^211^At reaction at the Cyclotron and PET Unit at Rigshospitalet in Copenhagen, Denmark. Preparation of free ^211^At was performed according to Lindegren et al. [23]. The CRC-15R dose calibrator ion chamber (Capintec, Inc., Ramsey, NJ, USA) was used to measure ^211^At activity concentrations of stock solutions prior to injection.

### Estimation of absorbed dose

Calculation of organ-specific mean absorbed doses (${\overline{\boldsymbol{D}}}_{\mathrm{organ}}$) was performed according to the Medical Internal Radiation Dose (MIRD) formalism, assuming homogeneous activity distribution within each organ [24].

$$ {\overline{\boldsymbol{D}}}_{\mathrm{organ}}=\frac{{\tilde{\boldsymbol{A}}}_{\mathrm{organ}}\times {\displaystyle {\sum}_{\boldsymbol{i}}}{\boldsymbol{n}}_{\boldsymbol{i}}{\boldsymbol{E}}_{\boldsymbol{i}}{\boldsymbol{\varPhi}}_{\boldsymbol{i}}}{{\boldsymbol{m}}_{\mathrm{organ}}}, $$

where *n*_*i*_ is the yield for radiation *i* with energy *E*_*i*_ and absorbed fraction *Φ*_*i*_ in the target organ with mass *m*_organ_.

Organ-specific biodistribution data for ^211^At between 0 and 24 h was taken from literature [8]. The biodistribution was assumed invariant from 24 h to 7 days. The cumulated activity *Ã*_organ_ was estimated from 0 h to 7 days using the trapezoidal rule. Dose contributions were only considered from α-particles emitted by ^211^At and its daughter polonium-211 (^211^Po). For all investigated tissues, the absorbed fraction was set to 1 due to the short mean range of emitted alpha particles [25].

### Animal experiments

**Figure 1:**
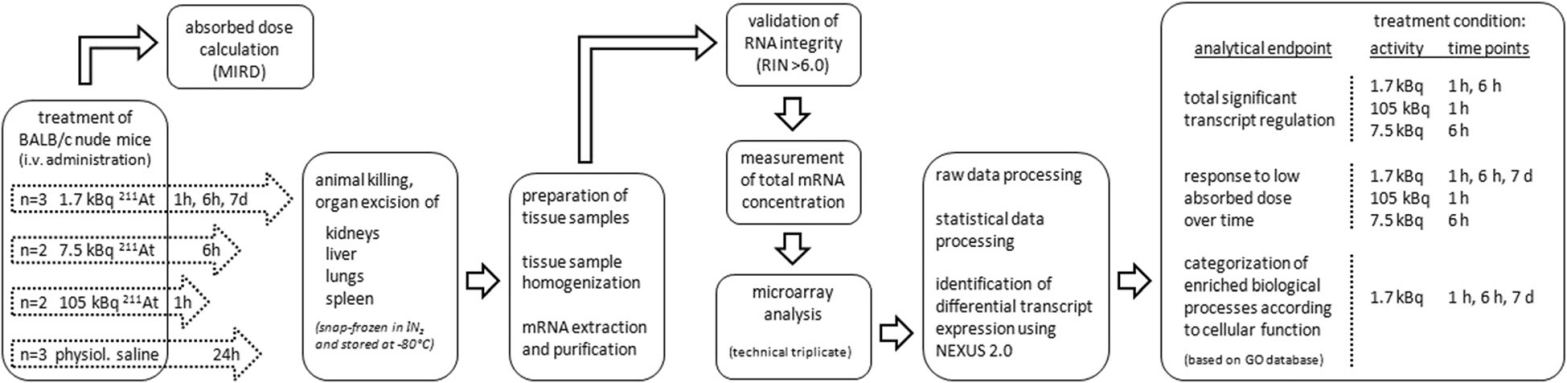
**Workflow diagram for overview on treatment and sampling.** Female BALB/c nude mice were intravenously injected with 1.7 kBq ^211^At and killed after 1 h, 6 h, and 7 days or injected with 105 or 7.5 kBq ^211^At and killed after 1 and 6 h, respectively. The kidneys, liver, lungs, and spleen were excised, frozen and stored. Kidney cortex and kidney medulla were dissected and treated separately. Total RNA was extracted from tissue samples and subjected to microarray analysis. For details on data processing and analysis, please refer to text.

Adult female BALB/c nude mice (CAnN.Cg-Foxn1nu/Crl, Charles River Laboratories International, Inc., Salzfeld, Germany) were injected into the tail vein with 1.7 kBq ^211^At in physiological saline solution (*n* = 3/group). The control group was mock-treated with phosphate buffered saline (*n* = 3). At 1 h, 6 h, and 7 days after injection, animals were anesthetized with sodium pentobarbital and killed via cardiac puncture. The control group was killed after 24 h. The kidneys, liver, lungs, and spleen were excised, flash-frozen, and stored at −80°C until analysis. Cortical and medullary kidney tissues were dissected and prepared separately. In addition, two groups with two mice each were treated as described above but injected with 105 or 7.5 kBq ^211^At to deliver 1.4 Gy to the thyroid over 1 and 6 h, respectively, and treated as described above. Please see workflow diagram for overview on treatment and sampling (Figure 1). All animal procedures were approved by the Ethical Committee on Animal Experiments in Gothenburg, Sweden.

### Gene expression analysis

Total RNA was extracted from individual tissue samples and concentration and integrity values were determined as described previously [20]. All samples were validated for subsequent analysis with a RIN value of at least 6.0. RNA samples were analyzed on Illumina MouseRef-8 Whole-Genome Expression BeadChips (Illumina; San Diego, CA, USA) using technical triplicates and processed at the Swegene Center for Integrative Biology Genomics DNA Microarray Resource Center (SCIBLU; Lund, Sweden). Image acquisition, raw signal quantification, data preprocessing, and quantile normalization were performed with Illumina and BioArray Software Environment (BASE) (SCIBLU) software as described previously [20]. Subsequent data processing was performed with Nexus Expression 2.0 (BioDiscovery; El Segundo, CA, USA) as described elsewhere [26].

The false discovery rate for differentially expressed transcripts was controlled according to the Benjamini-Hochberg method [27] with an adjusted *P* value cutoff of less than 0.01 and with a threshold of a 1.5-fold change or higher. Enriched Gene Ontology (GO) terms associated with a gene set were identified with a *P* value cutoff of less than 0.05. The GO database (http://www.geneontology.org) was used for analysis of associated GO terms and biological processes [28]. Enriched biological processes were categorized based on GO terms to establish comprehensive regulatory profiles according to cellular function as previously presented [20]. The intensity of response was expressed as the percentage of scored vs. the filtered number of transcripts calculated for all biological processes grouped in a (sub)category. Please see workflow diagram for overview on sample processing analytical endpoints (Figure 1). The gene expression data in this study have been deposited in NCBI's Gene Expression Omnibus (GEO accession GSE56894).

## Results

### Organ-specific absorbed doses

Administration of 1.7 kBq ^211^At solutions resulted in very low to low mean absorbed doses in the liver, lungs, spleen, and kidney tissues over 1 week (Table 1). One hour after injection, mean absorbed dose was lowest in the liver with 0.23 mGy and highest in the lungs with 1.9 mGy. One week after injection, the mean absorbed dose ranged from 6.3 to 50 mGy for the investigated tissues. The mean absorbed dose to the lungs was approximately threefold higher than to the spleen, sixfold higher than to the kidneys, and eightfold higher than to the liver. Compared with 1.7 kBq ^211^At, administration of 105 and 7.5 kBq ^211^At resulted in approximately 60-fold higher mean absorbed doses over 1 h and approximately fourfold higher mean absorbed doses over 6 h, respectively. In thyroid tissue, absorbed dose was around 78-, 127-, and 184-fold higher after 1 h, 6 h, and 7 d, respectively, compared with kidney tissue (data not shown). Please note that analysis of transcriptional regulation in thyroid in response to ^211^At is part of an ongoing study.

**Table 1:** **Tissue-specific absorbed doses**

	**Mean absorbed dose (mGy)**				
^211^ **At activity (kBq)**	**1.7**	**105**	**1.7**	**7.5**	**1.7**
**Time point**	**1 h**	**1 h**	**6 h**	**6 h**	**7 days**
Kidney cortex	0.29	18	2.6	11	7.6
Kidney medulla	0.29	18	2.6	11	7.6
Liver	0.23	14	2.0	8.9	6.3
Lungs	1.9	116	16	72	50
Spleen	0.86	53	5.7	25	14
Thyroid	23	1,400	320	1,400	1,800

Mean absorbed dose (mGy) to mouse tissues at the time of animal killing after intravenous administration of 1.7, 7.5, or 105 kBq ^211^At. Calculations are based on the Medical Internal Radiation Dose (MIRD) formalism [24] with biodistribution data reported by Garg et al. [8].

### Total transcriptional responses

**Figure 2:**
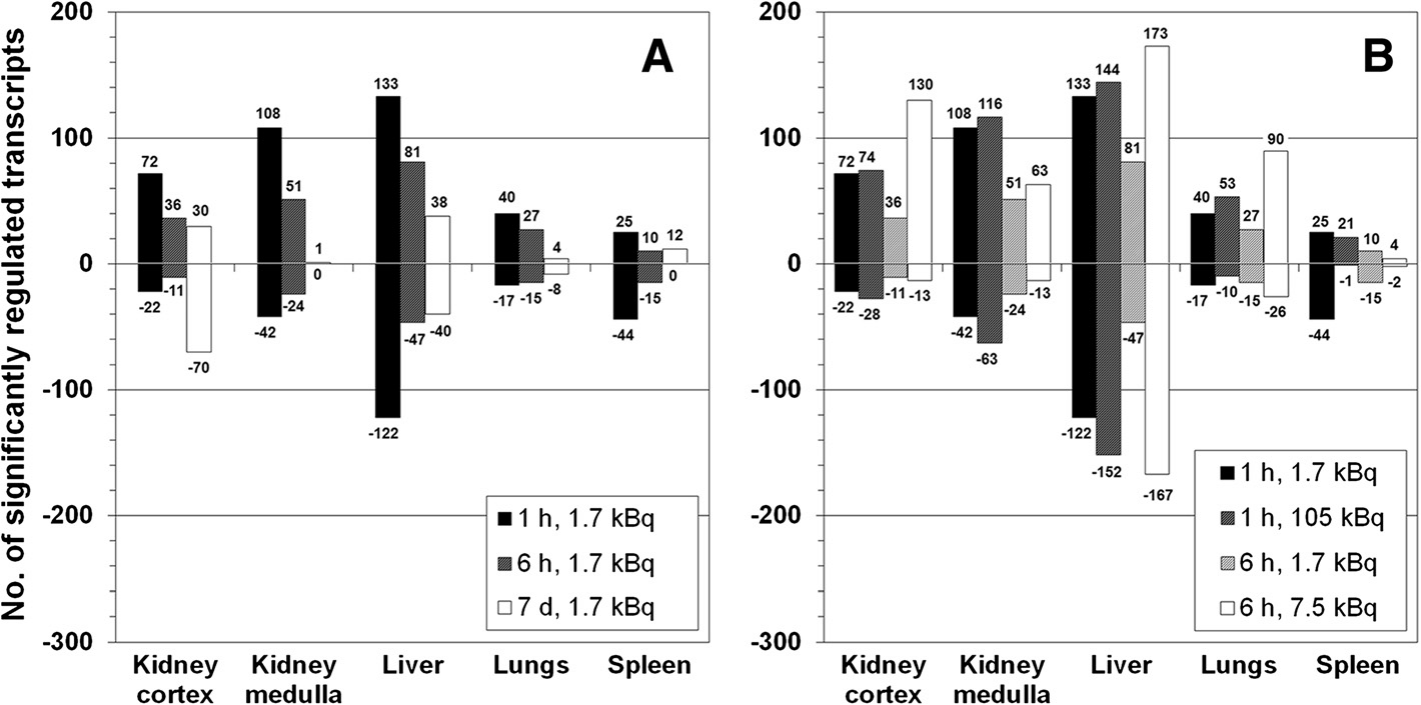
**Number of significantly regulated transcripts.** Comparison of the total number (no.) of significantly upregulated (positive numbers) and downregulated (negative numbers) transcripts in the kidney cortex and medulla, liver, lungs, and spleen. **(A)** shows responses at various time points after i.v. administration of 1.7 kBq ^211^At. **(B)** shows total transcript regulation at early time points dependent on dose rate, i.e., effects from 1.7 kBq compared to either 105 kBq ^211^At after 1 h or to 7.5 kBq ^211^At after 6 h.

The number of significantly upregulated transcripts from 1 h to 7 days in response to 1.7 kBq ^211^At varied between 1 and 133 in the tissues (Figure 2A). Between 0 and 122 transcripts were downregulated. Upregulation dominated over downregulation in two-thirds of all instances. The lowest number of regulated transcripts was observed in the kidney cortex after 6 h and in the other tissues after 7 days. In most tissues, an increased number of regulated transcripts - although marginal for some instances - could be seen with increased absorbed dose (rate) at 1 and 6 h (Figure 2B). However, spleen tissue showed an inverse response with a distinctly reduced number of significantly regulated transcripts at the higher absorbed dose (rate) at both time points (Figure 2B). Moreover, kidney medulla and spleen showed an increased transcript regulation at the higher dose rate (105 kBq administration, 1 h) than at the lower dose rate (7.5 kBq administration, 6 h). In contrast, the kidney cortex, liver, and lungs responded more strongly to the lower dose rate.

### Potential dose rate-sensitive molecular biomarkers

**Figure 3:**
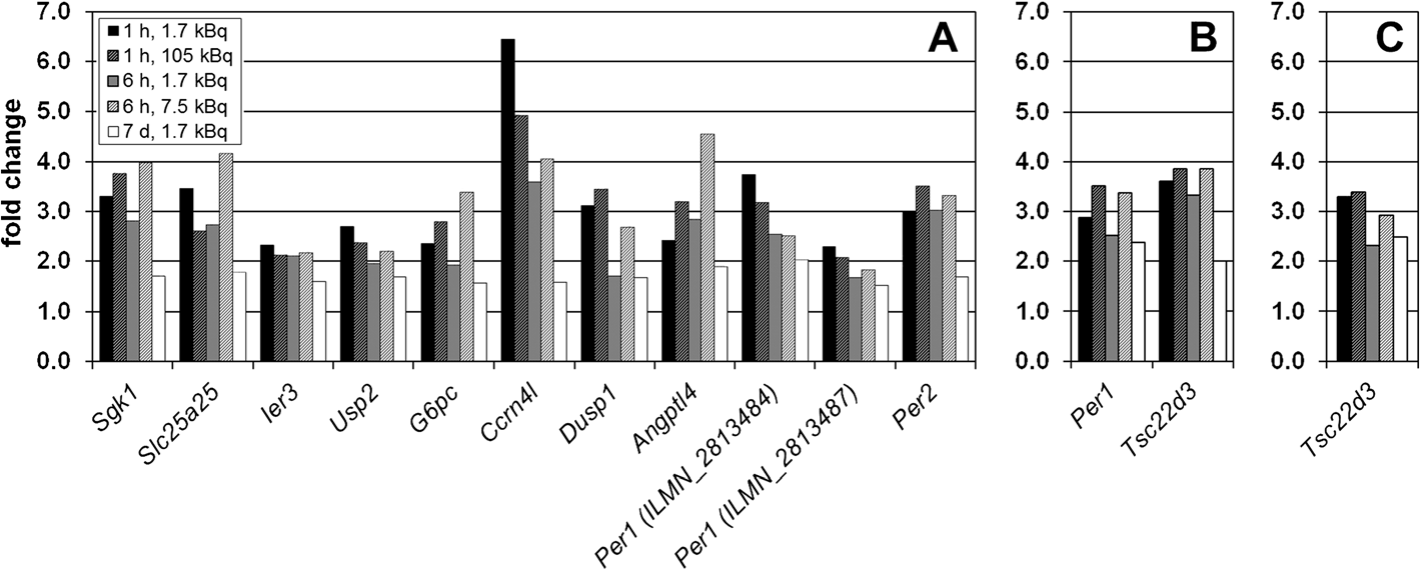
**Potential molecular biomarkers in the kidney cortex, lungs, and spleen for i.v.** ^**211**^ **At administration.** Differentially regulated genes on the transcriptional level responding at all investigated time points in the kidney cortex **(A)**, lungs **(B)**, and spleen **(C)** after injection of 1.7, 7.5, or 105 kBq ^211^At. Note the difference in scaling of the y-axis compared with Figure 4.

**Figure 4:**
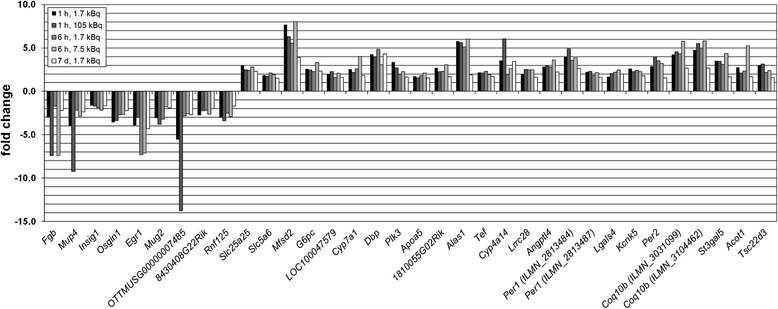
**Potential molecular biomarkers in the liver for i.v.** ^**211**^ **At administration.** Differentially regulated genes on the transcriptional level responding at all investigated time points in the liver after injection of 1.7, 7.5, or 105 kBq ^211^At. Note the difference in scaling of the y-axis compared with Figure 3.

The sample cohort was investigated for potential molecular biomarkers with sensitivity in the (very) low absorbed dose range. In addition, the dose rate effect for responses after 1 and 6 h was also analyzed. For the majority of transcripts regulated at all exposure conditions, fold change values were lowest after 7 days (Figures 3 and 4). In kidney cortex, ten genes were significantly regulated at all exposures, including two probe variants for the *Per1* gene (Figure 3A), all of which were continuously upregulated. In the lungs and spleen, only two and one gene(s) were significantly regulated at all exposures, respectively (Figure 3B, C). Similar to kidney cortex, neither the lungs nor spleen showed downregulation among commonly regulated genes. Liver tissue showed the highest yield with 32 significantly regulated genes at all exposures, including two probe variants for the *Coq10b* and *Per1* genes (Figure 4). Among these 34 transcripts, 25 were upregulated and all transcripts showed no change in direction of regulation with changing exposure condition. In kidney medulla, no genes were significantly regulated at all exposures within this sample cohort.

Among significantly regulated transcripts, the expression profile of potential molecular biomarkers is preferably a function of monotonous increase or decrease with absorbed dose/dose rate. As such, fold change values should be either higher or lower for increased activity at both time points compared to 1.7 kBq with no change in intensity of up- or downregulation relative to 1.7 kBq between time points. In kidney cortex, the *Sgk1*, *G6pc*, *Dusp1*, *Angptl4*, and *Per2* genes showed this pattern for potential biomarkers. In the lungs, the *Per1* and *Tsc22d3* genes also displayed this pattern. Although *Tsc22d3* showed the same pattern in spleen, the difference in fold change values between activities at 1 h was comparatively low. In the liver, 50% of the genes displayed this pattern, i.e., *Fgb*, *Mup4*, *Insig1*, *Osgin1*, *Egr1*, *Rnf125*, *Slc5a6*, *LOC100047579*, *Dbp*, *Cyp4a14*, *Angptl4*, two probe variants for *Per1*, *Lgals4*, *Kcnk5*, two probe variants for *Coq10b*, and *Tsc22d3*. It should be noted that differences in fold change within this pattern, i.e., between 1.7 kBq administrations compared to 105 kBq after 1 h or 7.5 kBq after 6 h, respectively, varied strongly between time points, genes, and tissues. In this regard, *Per1* (Illumina probe ID ILMN_2813484) in kidney cortex and *Tef* in liver showed this regulatory pattern, but differences in fold change values between both activities were below 3% at 1 and 6 h, respectively.

### Response profiles of biological processes

**Figure 5:**
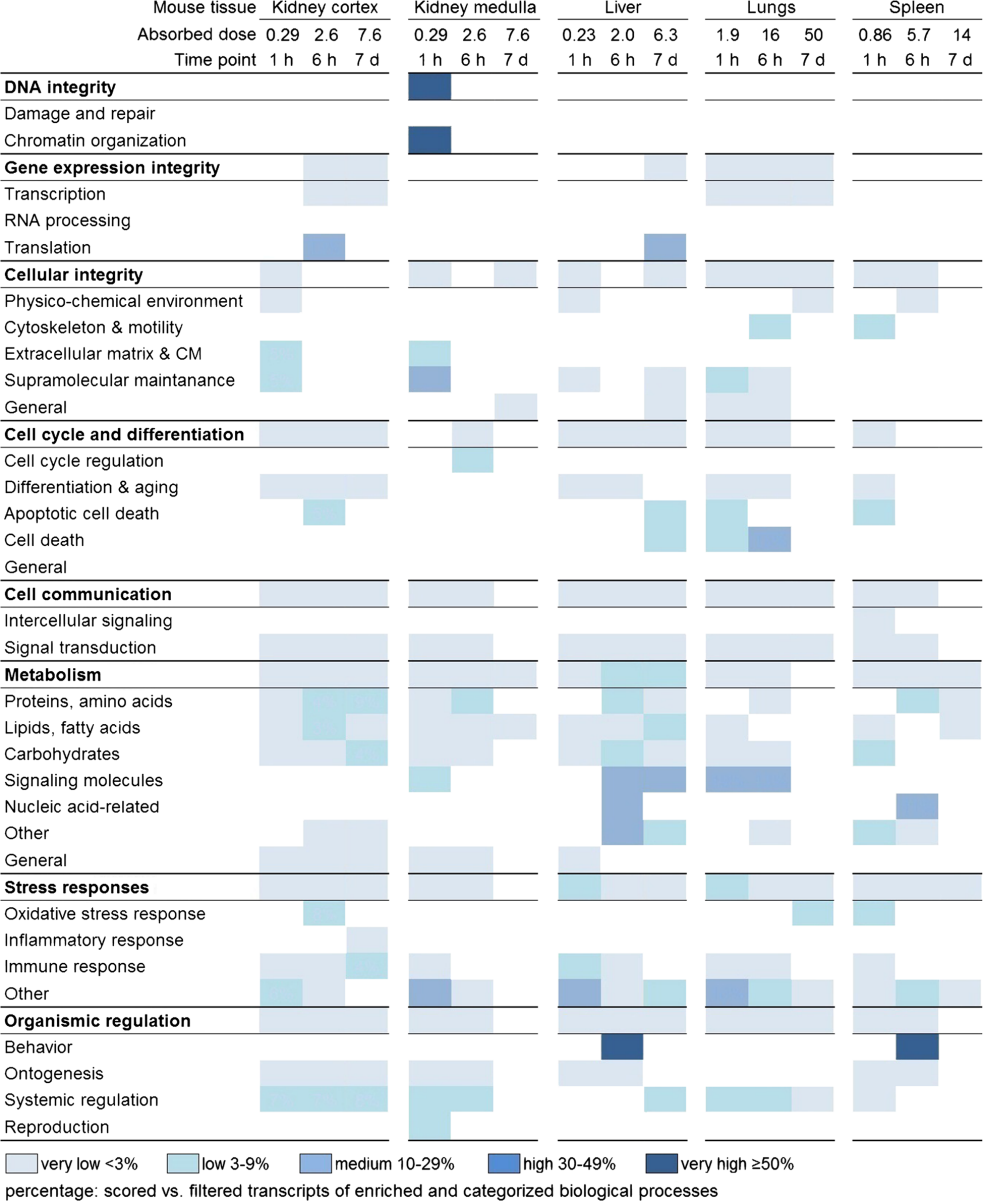
**Response profiles of enriched biological processes categorized after cellular function.** Significantly regulated transcripts were enriched for biological processes which were grouped in respective categories and subcategories of higher level cellular function. The percentage of scored vs. filtered transcripts is illustrated as very low <3%, low 3% to 9%, medium 10% to 29%, high 30% to 49%, and very high ≥50% and presented as very light blue, light blue, blue, dark blue, and very dark blue, respectively. Further information on categorized biological processes and absolute transcript numbers are presented in Additional file 1: Table S1, Additional file 2: Table S2, Additional file 3: Table S3, Additional file 4: Table S4, and Additional file 5: Table S5 for the kidney cortex and medulla, liver, lungs, and spleen, respectively. Absorbed dose received from 1.7 kBq ^211^At i.v. administration during respective time frame.

Transcriptional response to 1.7 kBq ^211^At was investigated on the level of cellular function based on established categorization of enriched biological processes (Figure 5). Complete lists of categorized biological processes are provided for all tissues in Additional file 1: Table S1, Additional file 2: Table S2, Additional file 3: Table S3, Additional file 4: Table S4, and Additional file 5: Table S5. Response patterns within subcategories of biological processes indicated tissue specificity. A common trend in regulatory intensity was seen in the tissues, with the vast majority of regulation incidences ranging from very low (below 3%) to low (from 3% to 9%). No response was observed for DNA damage and repair processes in any tissue, while regulation of chromatin organization occurred only at 1 h in kidney medulla. Gene expression integrity showed no regulation in either kidney medulla or spleen at any time point. Response to maintaining cellular integrity was more pronounced than for maintaining DNA or gene expression integrity in all tissues, although regulatory patterns differed between all tissues. Several subcategories in cell cycle and differentiation were not regulated in an individual tissue, yet no specific subcategory was void of responses in all tissues. In cell communication, signal transduction was affected in every tissue for all or most exposure conditions, while intercellular signaling was only affected after 1 h in spleen. Metabolism was diversely regulated in all tissues. The strongest overall response was seen in liver, followed by kidney cortex and medulla. Stress responses were observed in all tissues with general dominance of immune responses and unspecific responses (*Other*). In the category of organismic regulation, regulation incidences for behavior were not observed in both kidney tissues and lungs and neither for ontogenesis in the lungs nor for reproduction in the kidney cortex, liver, lungs, and spleen.

## Discussion

The synthetic radiohalogen ^211^At and the ^211^Po daughter emit two main α-particles, i.e., 5.87 and 7.45 MeV, which have an approximated range of 48 and 70 μm in liquid water, respectively [25]. Alpha particles are densely ionizing and cause non-homogeneous energy deposition within cells or tissues. The degree of homogeneity in cellular irradiation - i.e., the fraction of non-hit, single-hit, or multi-hit cells in a population - most likely influences the biological response of a given tissue. This response depends not only on the amount of administered activity and tissue-specific uptake and clearing rates but also on, e.g., organ morphology, tissue structure, and size and density of cells ([29], Josefsson A, Forssell-Aronsson E. Microdosimetric analysis of the radio halogens ^123^I, ^124^I, ^125^I, ^131^I and ^211^At, Submitted). Non-homogeneous irradiation may result in non-linear response to absorbed dose on the tissue level and/or increased intensity of, e.g., non-targeted effects [30]. Microdosimetric considerations of ^211^At exposure *in vivo* and the relevance of non-homogeneous irradiation at very low to low absorbed doses have been discussed previously in a related study [20]. Accordingly, very low to low absorbed doses in kidney, liver, lungs, and spleen tissues corresponded to non-homogeneous irradiations with large fractions of non-hit cells. With increased absorbed dose, the fraction of single-hit and multi-hit cells increases. In this context, it should be pointed out that genome-wide transcriptional responses were obtained from homogenized tissue samples. The presented data gives information on significant biological responses within tissue-specific cell populations in an *in vivo* context. Nevertheless, the non-homogeneous nature of α-particle exposure at (very) low absorbed doses from ^211^At introduces the question how much of the significant transcript response originated from the (single or multi) hit fraction(s) and to what extent the non-hit fraction contributed to the observed effects in each tissue.

The total number of significantly regulated transcripts in response to 1.7 kBq ^211^At varied with time and tissue type. Previous studies demonstrated that differential transcript expression in thyroid and non-thyroid tissues varied with ^211^At activity at 24 h after administration [19,20]. Taken together, these findings substantiate for the *in vivo* setting that ionizing radiation-induced responses strongly depend on exposure condition and time course. Recently, Heinonen and colleagues estimated differential expression time periods after stress exposure using a novel Bayesian likelihood ratio test [31]. Among others, the group measured transcript profiles of 77 differentially expressed genes in primary human endothelial cells *in vitro* after external irradiation of 2 Gy with a ^137^Cs source, demonstrating that the maximum differential expression (>1.5-fold change) occurred between 8 and 12 days after irradiation. Time-dependent change in differential expression constitutes an important factor in transcriptional analysis and, specifically, biomarker discovery. In the present study using internal radionuclide exposure, differences in differential expression between time points were influenced not only by, e.g., the biological response time of certain regulatory events but also by continuous irradiation and increased absorbed dose over respective periods. Hence, it is unfeasible to analyze time between exposure and effect as an individual factor in this setting. An external irradiation setup would be required to allow for both singular and continuous radiation exposure in order to differentiate between biological response time and (extent of) induced regulation.

In the dose-rate study, injected activities of 105 and 7.5 kBq were chosen so that the thyroid gland would receive a mean absorbed dose of 1.4 Gy over 1 and 6 h, respectively. Since the thyroid is a regulatory organ that is presumed to have a potential impact on non-thyroid tissue responses [20], we chose this setup to keep absorbed dose in thyroid tissue constant over both time periods. This would constrict the variable of absorbed dose dependence in the systemic setting of a regulatory organ impacting target organs. Concerning non-thyroid tissues, tissue-specific variations in ^211^At uptake and clearing rates did not give exact matching of mean absorbed dose within this setup, but respective mean absorbed doses lay at similar low levels. Nevertheless, transcript regulation showed sensitivity to differential dose rate exposure in this setup for all tissues. These regulatory responses also showed pronounced differences compared with thyroid tissue, which is subject of an ongoing study.

An inverse dose rate effect on total transcript regulation was observed in the kidney cortex, liver, and lungs. The inverse dose rate phenomenon has been demonstrated for different exposure conditions and biological end points, for instance for mutation induction in human lymphoblasts in response to ^137^Cs γ-rays [32], deletions size in the *HPRT* locus in human lymphoblastoid cells following 200 keV X-ray exposure [33], micronuclei induction in Lewis lung carcinoma cells exposed to ^60^Co γ-rays [34], and cytokine gene expression in human glioblastoma cell lines irradiated with ^137^Cs γ-rays [35]. A cell cycle-dependent phase of sensitivity has been proposed to explain the inverse dose rate effect, meaning that increased irradiation time at decreased dose rate increases the probability of a cell to enter a sensitive phase during cell-cycle progression [36,37]. A saturable intermediate state has been hypothesized as well, which postulates that an increase in probability for damage induction would not increase proportionally with increased number of hits to a cell [37,38]. Brenner and colleagues concluded in their modeling approach using the linear-quadratic + resensitization formalism that ‘all potential explanations of inverse dose rate effects predict that, at appropriately low doses, no dose rate effects of any kind are expected’ [38]. This statement provokes the question on the nature of the inverse dose rate effects observed for differential transcript expression at the (very) low absorbed doses from α-radiation observed in this study. Assuming relatively homogeneous tissue morphology and radionuclide distribution, one out of one thousand cells is estimated to be hit in the mGy range with very low probability for more than one hit per cell. Since the largest amount of cells contributing to expression data are within the non-hit fraction under these exposure conditions, complex non-targeted effects may contribute to non-linearity of responses. Furthermore, the relatively high LET value and biological effectiveness of ^211^At-emitted α-particles may increase the inverse dose rate effectiveness factor compared with γ-rays and X-rays. It is unclear at this point whether the transcripts that exhibited an inverse dose rate effect are regulated as an immediate response to particle hits or if their regulation is determined by regulatory networks in dependence of additional factors. The finding that dose rate effects differed between tissues implies an additional degree of complexity for radiation responses to (very) low absorbed doses or dose rates *in vivo*. Biological response on the organismic level needs to be understood in a dynamic context where some tissues would respond more intensively at a given time point or to a certain dose rate, while other tissues would show a less pronounced response; yet this relation might change or invert over time and with regard to dose rate.

A biomarker for ionizing radiation exposure should be indicative of a certain radiation-induced effect, such as molecular damage to the DNA or impaired cellular function. An ideal biomarker should be easily quantifiable and constitute a molecule (or a group of molecules) that changes in concentration or is modified upon exposure. The response should change monotonously with absorbed dose and occur early upon irradiation and remain detectable after a long time period. Sensitivity over a wide absorbed dose range is desirable but not mandatory, since a panel of biomarkers could be assorted covering a wider range. A biomarker should be sensitive to low dose rate and, ideally, indicate low, medium, or high dose rate exposure with, e.g., changes in time-of-onset and intensity of response. Transcriptional regulation responds to a stressor in a sensitive manner and continuous dysregulation of transcript expression can be expected to manifest itself earlier than detrimental changes on the protein level. This is an advantage of transcriptional biomarkers compared to protein biomarkers or general markers of tissue function. In a low dose exposure setting, an acute radiation response and related tissue damage are not expected, as opposed to an increased risk for cancerogenesis or latent damage. Correlation between early effects upon exposure and long-term health effects requires substantial knowledge of overall transcriptomic responses and individual gene regulation patterns. This approach can be used to establish risk estimation based on absorbed dose or, in critical cases, justify tissue biopsies of risk organs.

In this study, we investigated the up- or downregulation of transcript expression for the discovery of individual molecular biomarkers. BALB/c nude mice were chosen for these experiments since *in vivo* models for radionuclide therapy require immunodeficient mice to establish human tumor xenografts. The presented results should be applicable in that analytical context when studying normal tissue effects of radiolabeled agents in tumor-bearing mice. In this context, it should be noted that reduced expression of the DNA-dependent protein kinase catalytic subunit (DNA-PKcs) and single nucleotide polymorphisms (SNPs) in *Prkdc* have been reported for several BALB/c strains which affected DNA repair proficiency upon ionizing radiation exposure [39-43]. To the best of our knowledge, the specific strain used in this study has not been categorized regarding DNA repair deficiency, but it is assumed that the genetic background exhibits similarly increased radiation sensitivity. Accordingly, the observed responses can be related to radiation sensitive individuals in a clinical setting.

For the kidney cortex, liver, lungs, and spleen, several genes were identified that followed a potentially indicative pattern across all exposures, i.e., a consistent regulatory change between lower and higher injected activity at 1 and 6 h with either a direct or inverse dose rate effect - while showing a consistent trend relative to 1.7 kBq over time. Several of these genes were shared between tissues: angiopoietin-like protein 4 (*Angptl4*) was regulated in both the kidney cortex and liver and was nearly regulated in all tissues and at all mean absorbed doses (0.064 to 42 kBq ^211^At) after 24 h in the previous study [20]. The *Angptl4* gene product is thought to modulate vascular activity and tumor cell motility and invasiveness, which implies its significance for tumor progression and metastasis [44-46]. The prognostic value of *Angptl4* deregulation in ionizing radiation-induced cancerogenesis is promising, but accumulating evidence suggests that the function of *Angptl4* highly depends on, e.g., proteolytic processing and posttranslational modifications and thus can have opposite effects on vascular permeability in different cancers [47,48]. The versatile functions of *Angptl4* impede further speculation on long-term effects after ^211^At exposure, or ionizing radiation in general, at this point. Long-term studies are needed in order to evaluate whether or not the observed response is indicative of radiation-induced tumorigenesis or carcinogenesis at these early time points or if transcriptional regulation of *Angptl4* responds in a different context.

Either *Per1* or *Per2* (periodic clock genes 1 and 2) were commonly regulated in the kidney cortex, liver, and lungs at all exposure conditions. In our previous study on non-thyroid tissues, *Per1* also showed significant upregulation in these tissues after 24 h in an absorbed dose range from several mGy to around 1 Gy, i.e., specifically after administration of 0.64, 14, and 42 kBq ^211^At (data not shown in Langen et al. [20]; please refer to GEO:GSE40806). In thyroid, *Per1* was also consistently upregulated after 24 h at these injected activities, i.e., 0.5, 11, and 32 Gy, respectively (see supplemental material of Rudqvist et al. [19]). *Per2*, on the other hand, was significantly regulated only in liver and thyroid after 24 h in the low injected activity range. In both tissues, *Per2* was consistently downregulated with a similarly low differential expression ranging between −1.7 and −2.2-fold change. Taken together, we demonstrated that *Per1* and *Per2* responded differently in different tissues and across a wide-absorbed dose regimen from mGy to over 32 Gy. *Per* genes encode for negative regulators in the circadian feedback loop, thus regulating metabolism as well as various other cellular processes and circadian-dependent gene expression [49]. Disturbance of the circadian clock has been linked to cancer; specifically, deregulation of *Per1* and *Per2* have been connected to gastric cancer and suggested as prognostic markers [49-51]. Moreover, long-term effects in tumor suppression by Per proteins have been demonstrated in context with ionizing radiation exposure [52]. At a sub-lethal dose of 4 Gy γ-radiation, *mPer2* mutant mice showed graying of the coat after approximately 2 months and/or developed lymphoma after 5 months at a significantly increased rate compared with wild-type mice [52]. Consequently, disruption of *Per* gene expression can be expected to dampen - if not abolish - impede this protective feature and result in increased cancer risk. Whether responses in *Per1* and *Per2* are indicative of radiation-induced malignancies or if their regulation is mainly affected by metabolism and circadian rhythm needs to be addressed in further studies allowing for strict separation of these factors.

*Tsc22d3* (TSC22 domain family, member 3) was upregulated in the liver, lungs, and spleen at all treatment conditions. This finding was in agreement with a study by Koike and colleagues demonstrating upregulation of *TSC22* in normal human epidermal keratinocytes after 4 and 8 h following exposure to 10 Gy X-ray radiation [53]. In our previous study, *Tsc22d3* did not exhibit significant regulation after 24 h in liver or kidney tissues, and only few regulation incidences in lungs and spleen (data not shown in Langen et al. [20]; please refer to GEO:GSE40806). *Tsc22d3* was also not significantly regulated after 24 h in thyroid across an absorbed dose range from 0.05 to 32 Gy (see supplemental material of Rudqvist et al. [19]). In comparison with the aforementioned *Per* transcripts, *Tsc22d3* appeared to respond in a more restrictive fashion. The expressed protein shares sequence similarities with leucine zipper proteins, implying a function as a transcription factor [54]. Regulation of *Tsc22d3* appears to have a key function in anti-inflammatory and immunosuppressive effects of glucocorticoids and interleukin-10 [55]. Furthermore, interactions between *Tsc22D3* and transcription factors nuclear factor NF-kappa-B p105 subunit (NFKB1) and nuclear factor NF-kappa-B p100 subunit (NFKB2) have been demonstrated [55]. The knowledge base, however, is still scarce regarding long-term effects of *Tsc22d3* deregulation, and prognosis of late effects from ^211^At-induced upregulation cannot be made at this point. In a broader sense, even robust molecular biomarkers would underlie regulation networks, which would render extrapolation of effects over time difficult. Hence, observed responses in individual genes should be considered in the broader context of regulatory networks within a cell and in the larger context of tissues and organs within the body.

Cellular function (the quality of transcriptional effects) was characterized according to enriched transcript-associated biological processes. The response patterns differed between tissues at similar absorbed dose level - specifically comparing kidney cortex, kidney medulla, and liver - which was in agreement with a study demonstrating that ionizing radiation-induced effects are not preprogrammed genetic responses but rather depend on tissue origin [56]. The major lesion type of ionizing radiation is considered to be the induction of DSB in the DNA molecule. The LET value of ^211^At-emitted α-particles is nearly 100 keV/μm with a high effectiveness of producing DSB [2,3], which should be considered in the analysis and interpretation of induced effects. In the present study, DNA damage and repair pathways did not show transcriptional response in enriched biological processes. A fold change value of at least 1.5 was chosen to exclude a large proportion of weakly responding genes and identify more pronounced changes in regulation, and accordingly, more suitable candidate genes (transcripts). In another research context, a somewhat lower fold change threshold might be used which would show more weakly responding genes (transcripts). Another factor that may lead to false-negative observation is data convolution from mixed cell populations, i.e., significant responses in a certain cell type may be dampened due to low cell type frequency. Nevertheless, when the DNA damage burden does not exceed the level of DNA damage recognition, transcriptional regulation of respective proteins is not expected. Accordingly, responses in DNA damage and repair processes may not be sensitive biomarkers in the low-dose regimen. However, chromatin organization was strongly affected after 1 h in kidney medulla and also showed response to at least one absorbed dose level after 24 h in all of the investigated tissues in the previous study [20]. Based on studies by Bakkenist and Kastan, chromatin organization was speculated to be a potential biomarker for ionizing radiation exposure [57]. The work indicated that activation of the ataxia-telangiectasia mutated (ATM) protein - a crucial player in DNA damage recognition - potentially results from structural changes of chromatin organization and may not strictly depend on direct protein-DNA binding [57]. In fact, responses in chromatin organization were detected in each of the investigated tissues after 24 h at certain (very) low absorbed doses from ^211^At as previously reported [20]. These findings suggest monitoring of biological processes for chromatin organization, i.e., respective key genes involved in process regulation, as potential *in vivo* biomarkers for (very) low absorbed doses of ionizing radiation. In this regard, chromatin organization may exhibit increased biomarker sensitivity for α-emitters compared to β-emitters due to the higher probability for producing DSB lesions per decay event.

## Conclusions

In conclusion, the quality and quantity of transcriptional responses to i.v. administration of 1.7, 7.5, or 105 kBq ^211^At in BALB/c nude mice were tissue-specific. Total transcript regulation after 1 h, 6 h, or 7 days showed dose rate dependency even in the (very) low absorbed dose range. Furthermore, the extent as well as relative increase or decrease in number of significantly regulated transcripts varied between time points and tissues. Categorization of enriched biological processes revealed diverse and tissue-specific regulation of cellular function after ^211^At administrations. *Angptl4*, *Per1* and *Per2*, and *Tsc22d3* showed the highest potential for being biomarkers of low-dose α-particle exposure. Identifying detrimental dysregulation of gene expression early after treatment is an essential part for counteracting toxic side effects in clinical practice. A deterioration of tissue function was not expected at the investigated time points after (very) low-dose exposure. In long-term studies, health effects such as decreased function or increased rate of cancerogenesis in respective tissues could be measured in parallel to genome-wide transcriptional analysis and correlated to the early transcriptional effects reported here.

Significant regulation of these transcripts, however, was not detected in every tissue, which may further indicate that tissue specificity is a relevant factor not only for ionizing radiation-induced cellular responses but also for robust biomarkers. Exposure of normal tissues to low-dose ionizing radiation is a critical parameter in radiation therapy. The relationship between early cellular responses and late organic outcomes is a relevant aspect for risk limitation and optimization of treatment planning. Further research on genome-wide transcript regulation *in vivo* is indispensable to build the knowledge base on normal tissue responses to ionizing radiation exposure.

## Additional files

Additional file 1: Table S1. Categorized biological processes in kidney cortex tissue. Additional file 2: Table S2. Categorized biological processes in kidney medulla tissue. Additional file 3: Table S3. Categorized biological processes in liver tissue. Additional file 4: Table S4. Categorized biological processes in lung tissue. Additional file 5: Table S5. Categorized biological processes in spleen tissue.
