# Supplementary material for: Transcriptional response in normal mouse tissues after i.v. 211At administration - response related to absorbed dose, dose rate, and time
Source: EJNMMI Res. 2015 Jan 28;5:1. doi: 10.1186/s13550-014-0078-7 (PMC4384707; doi:10.1186/s13550-014-0078-7)
Supplement: Additional file 2: Table S2. — Categorized biological processes in kidney medulla tissue. [file 13550_2014_78_MOESM2_ESM.docx]

**TABLE S2. Categorized biological processes in kidney medulla tissue**

|  |  |  |  |  |  |  |  |  |
| --- | --- | --- | --- | --- | --- | --- | --- | --- |
|  |  |  |  |  |  |  |  |  |
|  |  |  |  |  |  | ^211^At activity (kBq): | | |
| No. of filtered trancripts |  |  |  |  |  | 1.7 | 1.7 | 1.7 |
|  |  | **Category** | |  |  | Time point: | | |
|  |  |  | **Subcategory** | |  | 1 h | 6 h | 7 d |
|  |  |  |  | Enriched biological process |  | No. of scored transcripts | | |
| 1 |  | **DNA integrity** | | |  | **1** | **0** | **0** |
| 0 |  |  | **Damage and repair** | |  | **0** | **0** | **0** |
|  |  |  |  | *none* |  |  |  |  |
| 1 |  |  | **Chromatin organization** | |  | **1** | **0** | **0** |
|  |  |  |  | nucleosome spacing |  | 1 |  |  |
| 477 |  | **Gene expression integrity** | | |  | **0** | **0** | **0** |
| 468 |  |  | **Transcription** | |  | **0** | **0** | **0** |
|  |  |  |  | *none* |  |  |  |  |
| 1 |  |  | **RNA processing** | |  | **0** | **0** | **0** |
|  |  |  |  | *none* |  |  |  |  |
| 8 |  |  | **Translation** | |  | **0** | **0** | **0** |
|  |  |  |  | *none* |  |  |  |  |
| 703 |  | **Cellular integrity** | | |  | **11** | **0** | **1** |
| 184 |  |  | **Physico-chemical environment** | |  | **0** | **0** | **0** |
|  |  |  |  | *none* |  |  |  |  |
| 28 |  |  | **Cytoskeleton & motility** | |  | **0** | **0** | **0** |
|  |  |  |  | *none* |  |  |  |  |
| 40 |  |  | **Extracellular matrix & CM** | |  | **2** | **0** | **0** |
|  |  |  |  | negative regulation of cell adhesion |  | 2 |  |  |
| 86 |  |  | **Supramolecular maintanance** | |  | **9** | **0** | **0** |
|  |  |  |  | mitochondrial transport |  | 2 |  |  |
|  |  |  |  | protein folding |  | 6 |  |  |
|  |  |  |  | protein refolding |  | 1 |  |  |
| 365 |  |  | **General** | |  | **0** | **0** | **1** |
|  |  |  |  | lipid transport |  |  |  | 1 |
| 234 |  | **Cell cycle and differentiation** | | |  | **0** | **2** | **0** |
| 36 |  |  | **Cell cycle regulation** | |  | **0** | **2** | **0** |
|  |  |  |  | regulation of cell growth |  |  | 2 |  |
| 139 |  |  | **Differentiation & aging** | |  | **0** | **0** | **0** |
|  |  |  |  | *none* |  |  |  |  |
| 44 |  |  | **Apoptotic cell death** | |  | **0** | **0** | **0** |
|  |  |  |  | *none* |  |  |  |  |
| 15 |  |  | **Cell death** | |  | 0 | 0 | 0 |
|  |  |  |  | *none* |  |  |  |  |
| 0 |  |  | **General** | |  | 0 | 0 | 0 |
|  |  |  |  | *none* |  |  |  |  |
| 285 |  | **Cell communication** | | |  | **3** | **5** | **0** |
| 34 |  |  | **Intercellular signaling** | |  | **0** | **0** | **0** |
|  |  |  |  | *none* |  |  |  |  |
| 251 |  |  | **Signal transduction** | |  | **3** | **5** | **0** |
|  |  |  |  | progesterone receptor signaling pathway |  | 1 |  |  |
|  |  |  |  | two-component signal transduction system (phosphorelay) | | 2 | 2 |  |
|  |  |  |  | integrin-mediated signaling pathway |  |  | 2 |  |
|  |  |  |  | fibroblast growth factor receptor signaling pathway | |  | 1 |  |
| 593 |  | **Metabolism** | | |  | **6** | **11** | **1** |
| 46 |  |  | **Proteins, amino acids** | |  | **1** | **4** | **0** |
|  |  |  |  | glutamine biosynthesis |  | 1 | 1 |  |
|  |  |  |  | glycine metabolism |  |  | 1 |  |
|  |  |  |  | polyamine biosynthesis |  |  | 1 |  |
|  |  |  |  | protein catabolism |  |  | 1 |  |
| 246 |  |  | **Lipids, fatty acids** | |  | **2** | **4** | **1** |
|  |  |  |  | GPI anchor biosynthesis |  | 1 |  |  |
|  |  |  |  | negative regulation of lipoprotein lipase activity |  | 1 | 1 |  |
|  |  |  |  | lipoprotein metabolism |  |  |  | 1 |
|  |  |  |  | positive regulation of lipid metabolism |  |  | 1 |  |
|  |  |  |  | acetyl-CoA metabolism |  |  | 1 |  |
|  |  |  |  | acetyl-CoA biosynthesis from pyruvate |  |  | 1 |  |
| 70 |  |  | **Carbohydrates** | |  | **1** | **2** | **0** |
|  |  |  |  | glycerol biosynthesis from pyruvate |  | 1 | 1 |  |
|  |  |  |  | glycogen biosynthesis |  |  | 1 |  |
| 16 |  |  | **Signaling molecules** | |  | **1** | **0** | **0** |
|  |  |  |  | progesterone metabolism |  | 1 |  |  |
| 9 |  |  | **Nucleic acid-related** | |  | **0** | **0** | **0** |
|  |  |  |  | *none* |  |  |  |  |
| 34 |  |  | **Other** | |  | **0** | **0** | **0** |
|  |  |  |  | *none* |  |  |  |  |
| 172 |  |  | **General** | |  | **1** | **1** | **0** |
|  |  |  |  | S-adenosylmethionine biosynthesis |  | 1 |  |  |
|  |  |  |  | nitrogen compound metabolism |  |  | 1 |  |
| 364 |  | **Stress responses** | | |  | **9** | **1** | **0** |
| 24 |  |  | **Oxidative stress response** | |  | **0** | **0** | **0** |
|  |  |  |  | *none* |  |  |  |  |
| 44 |  |  | **Inflammatory response** | |  | **0** | **0** | **0** |
|  |  |  |  | *none* |  |  |  |  |
| 219 |  |  | **Immune response** | |  | **1** | **0** | **0** |
|  |  |  |  | regulation of interleukin-6 biosynthesis |  | 1 |  |  |
| 77 |  |  | **Other** | |  | **8** | **1** | **0** |
|  |  |  |  | ER overload response |  | 1 |  |  |
|  |  |  |  | response to heat |  | 2 |  |  |
|  |  |  |  | response to unfolded protein |  | 4 |  |  |
|  |  |  |  | skeletal muscle regeneration |  | 1 |  |  |
|  |  |  |  | cellular response to starvation |  |  | 1 |  |
| 335 |  | **Organismic regulation** | | |  | **8** | **9** | **0** |
| 2 |  |  | **Behavior** | |  | **0** | **0** | **0** |
|  |  |  |  | *none* |  |  |  |  |
| 224 |  |  | **Ontogenesis** | |  | **2** | **4** | **0** |
|  |  |  |  | positive regulation of striated muscle development | | 1 |  |  |
|  |  |  |  | somite specification |  | 1 |  |  |
|  |  |  |  | angiogenesis |  |  | 3 |  |
|  |  |  |  | cartilage condensation |  |  | 1 |  |
| 85 |  |  | **Systemic regulation** | |  | **5** | **5** | **0** |
|  |  |  |  | circadian rhythm |  | 2 | 2 |  |
|  |  |  |  | rhythmic process |  | 3 | 3 |  |
| 24 |  |  | **Reproduction** | |  | **1** | **0** | **0** |
|  |  |  |  | parturition |  | 1 |  |  |
