# Supplementary material for: Transcriptional response in normal mouse tissues after i.v. 211At administration - response related to absorbed dose, dose rate, and time
Source: EJNMMI Res. 2015 Jan 28;5:1. doi: 10.1186/s13550-014-0078-7 (PMC4384707; doi:10.1186/s13550-014-0078-7)
Supplement: Additional file 3: Table S3. — Categorized biological processes in liver tissue. [file 13550_2014_78_MOESM3_ESM.docx]

**TABLE S3. Categorized biological processes in liver tissue**

|  |  |  |  |  |  |  |  |  |
| --- | --- | --- | --- | --- | --- | --- | --- | --- |
|  |  |  |  |  |  |  |  |  |
|  |  |  |  |  |  | ^211^At activity (kBq): | | |
| No. of filtered trancripts |  |  |  |  |  | 1.7 | 1.7 | 1.7 |
|  |  | **Category** | |  |  | Time point: | | |
|  |  |  | **Subcategory** | |  | 1 h | 6 h | 7 d |
|  |  |  |  | Enriched biological process |  | No. of scored transcripts | | |
| 1 |  | **DNA integrity** | | |  | **0** | **0** | **0** |
| 0 |  |  | **Damage and repair** | |  | **0** | **0** | **0** |
|  |  |  |  | *none* |  |  |  |  |
| 1 |  |  | **Chromatin organization** | |  | **0** | **0** | **0** |
|  |  |  |  | *none* |  |  |  |  |
| 477 |  | **Gene expression integrity** | | |  | **0** | **0** | **2** |
| 468 |  |  | **Transcription** | |  | **0** | **0** | **0** |
|  |  |  |  | *none* |  |  |  |  |
| 1 |  |  | **RNA processing** | |  | **0** | **0** | **0** |
|  |  |  |  | *none* |  |  |  |  |
| 8 |  |  | **Translation** | |  | **0** | **0** | **2** |
|  |  |  |  | positive regulation of translation |  |  |  | 1 |
|  |  |  |  | regulation of translational initiation |  |  |  | 1 |
| 703 |  | **Cellular integrity** | | |  | **4** | **0** | **3** |
| 184 |  |  | **Physico-chemical environment** | |  | **2** | **0** | **0** |
|  |  |  |  | iron ion homeostasis |  | 2 |  |  |
| 28 |  |  | **Cytoskeleton & motility** | |  | **0** | **0** | **0** |
|  |  |  |  | *none* |  |  |  |  |
| 40 |  |  | **Extracellular matrix & CM** | |  | **0** | **0** | **0** |
|  |  |  |  | *none* |  |  |  |  |
| 86 |  |  | **Supramolecular maintanance** | |  | **2** | **0** | **2** |
|  |  |  |  | negative regulation of enzyme activity |  |  |  | 1 |
|  |  |  |  | protein processing |  | 2 |  |  |
|  |  |  |  | protein refolding |  |  |  | 1 |
| 365 |  |  | **General** | |  | **0** | **0** | **1** |
|  |  |  |  | maintenance of mitochondrion localization |  |  |  | 1 |
| 234 |  | **Cell cycle and differentiation** | | |  | **3** | **1** | **3** |
| 36 |  |  | **Cell cycle regulation** | |  | **0** | **0** | **0** |
|  |  |  |  | *none* |  |  |  |  |
| 139 |  |  | **Differentiation & aging** | |  | **3** | **1** | **0** |
|  |  |  |  | regulation of cell proliferation |  | 3 |  |  |
|  |  |  |  | regulation of fat cell differentiation |  |  | 1 |  |
| 44 |  |  | **Apoptotic cell death** | |  | **0** | **0** | **2** |
|  |  |  |  | negative regulation of apoptosis |  |  |  | 2 |
| 15 |  |  | **Cell death** | |  | **0** | **0** | **1** |
|  |  |  |  | negative regulation of non-apoptotic programmed cell death |  |  |  | 1 |
| 0 |  |  | **General** | |  | **0** | **0** | **0** |
|  |  |  |  | *none* |  |  |  |  |
| 285 |  | **Cell communication** | | |  | **2** | **3** | **4** |
| 34 |  |  | **Intercellular signaling** | |  | **0** | **0** | **0** |
|  |  |  |  | *none* |  |  |  |  |
| 251 |  |  | **Signal transduction** | |  | **2** | **3** | **4** |
|  |  |  |  | two-component signal transduction system (phosphorelay) |  | 2 | 2 |  |
|  |  |  |  | positive regulation of signal transduction |  |  | 1 |  |
|  |  |  |  | cytokine and chemokine mediated signaling pathway |  |  |  | 3 |
|  |  |  |  | regulation of MAPK activity |  |  |  | 1 |
| 593 |  | **Metabolism** | | |  | **8** | **21** | **26** |
| 46 |  |  | **Proteins, amino acids** | |  | **0** | **3** | **1** |
|  |  |  |  | L-cysteine catabolism to taurine |  |  | 1 |  |
|  |  |  |  | glycine metabolism |  |  | 1 |  |
|  |  |  |  | taurine metabolism |  |  | 1 |  |
|  |  |  |  | glutamine biosynthesis |  |  |  | 1 |
| 246 |  |  | **Lipids, fatty acids** | |  | **3** | **5** | **18** |
|  |  |  |  | acyl-CoA metabolism |  | 3 |  | 2 |
|  |  |  |  | cholesterol catabolism |  |  |  | 1 |
|  |  |  |  | lipid metabolism |  |  |  | 4 |
|  |  |  |  | cholesterol metabolism |  |  |  | 2 |
|  |  |  |  | long-chain fatty acid metabolism |  |  |  | 1 |
|  |  |  |  | retinol metabolism |  |  |  | 1 |
|  |  |  |  | ganglioside biosynthesis |  |  | 1 | 1 |
|  |  |  |  | ergosterol biosynthesis |  |  | 1 |  |
|  |  |  |  | negative regulation of lipoprotein lipase activity |  |  | 1 | 1 |
|  |  |  |  | positive regulation of fatty acid metabolism |  |  | 1 |  |
|  |  |  |  | positive regulation of lipid metabolism |  |  | 1 | 1 |
|  |  |  |  | vitamin D metabolism |  |  |  | 1 |
|  |  |  |  | steroid metabolism |  |  |  | 3 |
| 70 |  |  | **Carbohydrates** | |  | **2** | **6** | **1** |
|  |  |  |  | glycogen biosynthesis |  | 2 |  | 1 |
|  |  |  |  | glycerol metabolism |  |  | 2 |  |
|  |  |  |  | negative regulation of gluconeogenesis |  |  | 1 |  |
|  |  |  |  | glycerol biosynthesis from pyruvate |  |  | 1 |  |
|  |  |  |  | positive regulation of glucose import |  |  | 1 |  |
|  |  |  |  | response to glucose stimulus |  |  | 1 |  |
| 16 |  |  | **Signaling molecules** | |  | **0** | **2** | **3** |
|  |  |  |  | C21-steroid hormone biosynthesis |  |  | 2 |  |
|  |  |  |  | hormone biosynthesis |  |  |  | 1 |
|  |  |  |  | nitric oxide biosynthesis |  |  |  | 1 |
|  |  |  |  | positive regulation of nitric oxide biosynthesis |  |  |  | 1 |
| 9 |  |  | **Nucleic acid-related** | |  | **0** | **1** | **0** |
|  |  |  |  | uridine metabolism |  |  | 1 |  |
| 34 |  |  | **Other** | |  | **0** | **4** | **3** |
|  |  |  |  | glycosaminoglycan catabolism |  |  | 1 |  |
|  |  |  |  | Mo-molybdopterin cofactor biosynthesis |  |  | 1 |  |
|  |  |  |  | carboxylic acid metabolism |  |  | 1 |  |
|  |  |  |  | carotenoid biosynthesis |  |  |  | 2 |
|  |  |  |  | nitrogen compound metabolism |  |  |  | 1 |
|  |  |  |  | glycerol metabolism |  |  | 1 |  |
| 172 |  |  | **General** | |  | **3** | **0** | **0** |
|  |  |  |  | biosynthesis |  | 3 |  |  |
| 364 |  | **Stress responses** | | |  | **26** | **3** | **6** |
| 24 |  |  | **Oxidative stress response** | |  | **0** | **0** | **0** |
|  |  |  |  | *none* |  |  |  |  |
| 44 |  |  | **Inflammatory response** | |  | **0** | **0** | **0** |
|  |  |  |  | *none* |  |  |  |  |
| 219 |  |  | **Immune response** | |  | **16** | **1** | **0** |
|  |  |  |  | immune response |  | 14 |  |  |
|  |  |  |  | response to virus |  | 2 |  |  |
|  |  |  |  | regulation of interleukin-6 biosynthesis |  |  | 1 |  |
| 77 |  |  | **Other** | |  | **10** | **2** | **6** |
|  |  |  |  | response to unfolded protein |  | 5 |  | 2 |
|  |  |  |  | response to heat |  | 3 |  |  |
|  |  |  |  | cellular response to starvation |  | 2 |  | 2 |
|  |  |  |  | response to sterol depletion |  |  | 2 | 1 |
|  |  |  |  | ER overload response |  |  |  | 1 |
| 335 |  | **Organismic regulation** | | |  | **10** | **10** | **5** |
| 2 |  |  | **Behavior** | |  | **0** | **1** | **0** |
|  |  |  |  | behavior |  |  | 1 |  |
| 224 |  |  | **Ontogenesis** | |  | **2** | **1** | **0** |
|  |  |  |  | anagen |  | 2 |  |  |
|  |  |  |  | cartilage condensation |  |  | 1 |  |
| 85 |  |  | **Systemic regulation** | |  | **8** | **8** | **5** |
|  |  |  |  | rhythmic process |  | 5 | 5 | 3 |
|  |  |  |  | circadian rhythm |  | 3 | 3 | 2 |
| 24 |  |  | **Reproduction** | |  | **0** | **0** | **0** |
|  |  |  |  | *none* |  |  |  |  |
|  | | | | | | | | |
